# Assessing the Control of Postharvest Gray Mold Disease on Tomato Fruit Using Mixtures of Essential Oils and Their Respective Hydrolates

**DOI:** 10.3390/plants10081719

**Published:** 2021-08-20

**Authors:** Conny Brito, Henrik Hansen, Luis Espinoza, Martín Faúndez, Andrés F. Olea, Sebastián Pino, Katy Díaz

**Affiliations:** 1 Departamento de Ingeniería Química y Ambiental, Universidad Técnica Federico Santa María, Avenida España 1680, Valparaíso 2340000, Chile; connybritoescudero@gmail.com (C.B.); Henrik.hansen@usm.cl (H.H.); 2 Departamento de Química, Universidad Técnica Federico Santa María, Avenida España 1680, Valparaíso 2340000, Chile; luis.espinozac@usm.cl (L.E.); martin.faundez.12@usm.cl (M.F.); 3 Instituto de Ciencias Químicas Aplicadas, Facultad de Ingeniería, Universidad Autónoma de Chile, El Llano Subercaseaux 2801, Santiago 8900000, Chile; 4 LABSUN (Laboratorio Sustentable Natural), Valparaíso 2340000, Chile; sebastian.pino@alumnos.usm.cl

**Keywords:** antifungal activity, tomato, *Botrytis cinerea*, natural products, aromatic plants

## Abstract

Gray mold disease, which is caused by *Botrytis cinerea* Pers ex. Fr., results in serious economic losses to *Lycopersicum esculentum* (tomato) crop productivity. In this study, we explored the possibility that mixtures of essential oils (EOs) and their respective hydrolates (HYSs) could be used to control this disease. Thus, EOs and HYSs were obtained from *Origanum vulgare*, *Thymus vulgaris*, *Citrus limon*, and *Citrus sinensis* by hydrodistillation. In vitro antifungal activities were evaluated, and EC_50_ values of 15.9 and 19.8 µg/mL were obtained for EOs of thyme and oregano, respectively. These activities are due mainly to volatile compounds, thymol and carvacrol. Results from in vivo assays show that although most tomatoes were infested five days after inoculation, the damage was considerably reduced by the application of an EO/HYS mixture of thyme. The disease incidence indexes of *B. cinerea* tomato rot, percentage and severity, measured four days after inoculation, were reduced by 70% and 76%, respectively, as compared with the inoculum control. These results suggest that a combination of HYSs and EOs enhances antifungal activity, and that optimization of relative concentrations, volumes, and the nature of the compounds, could design a formulation able to control *B. cinerea* inoculum on tomato fruits.

## 1. Introduction

*Botrytis cinerea* is a phytopathogenic fungi, also known as “gray mold fungus”, that causes serious pre- and postharvest diseases in more than 200 different plant species, but considering other *Botrytis* spp., this number increases to 1500, including important food crops [1,2]. The gray mold disease affects almost every aspect of attacked plants during growth, storage, and transport of the final product. Therefore, the ubiquitous presence of *B. cinerea* results in significant economic losses to the horticulture and fruit industry, and it has been estimated that at least 20% of world crops are affected by *B. cinerea*, producing economic losses of approximately 10–100 million USD per year. Crop protection agents (CPAs), currently used to control *B. cinerea*, are mainly synthetic chemical compounds designed to prevent infection and to minimize postharvest losses [3]. Although these CPAs present a variety of modes of action against *B. cinerea*, the magnitude and frequency of fungicidal treatments have induced the appearance of resistant isolates of this fungus [4]. Thus, the economic importance of infected crops and appearance of resistant isolates has prompted the quest of new and efficient antifungal CPA. However, the negative public perception related to application of chemicals that could contaminate ground and natural water sources, has led this research to assess natural products with antifungal properties. In this approach, numerous plants have been studied and plant metabolites with various antibiotic, anticancer, and antifungal activities have been extracted, isolated, and characterized [5,6,7,8,9,10]. Then, the most active compounds have been chemically modified to obtain analogs to enhance their activities [11,12,13]. Different series of synthetic analogs have been evaluated against *B. cinerea* and results have been used to establish structure-activity relationships [14,15,16]. In addition, it has been known for many centuries that aromatic plants are a source of compounds possessing interesting properties for different applications. Complex mixtures of volatile compounds such as terpenes and terpenoids, generically known as essential oils (EOs), are obtained by extraction of aromatic plants, and so far, more than 3000 EOs have been identified and at least 300 have enormous economic importance due to their increased use in food, pharmaceutical, cosmetic, and perfume industries [17,18]. EOs are commonly obtained by steam distillation or hydrodistillation, and at the end of this process, hydrosols or hydrolates (HYSs) are obtained as an aqueous fraction that separates from the EOs [19]. GC-MS analysis and/or NMR spectroscopy have demonstrated that nonpolar molecules are the main components of EOs, whereas more hydrophilic molecules and traces of EOs form HYSs [20]. Thus, the physicochemical, and biological properties of both EOs and HYSs depend on the nature and relative concentration of their compounds [21,22]. Several studies have shown that EOs and HYSs exhibit biological activities, such as, cytotoxicity, antioxidant, antibacterial, and antifungal [23,24,25,26,27,28,29,30], which depend on the nature and relative concentration of their compounds. Interestingly, the activity of EOs seems to be higher than that of separate compounds, indicating the existence of a synergic effect [31,32,33]. Thus, much effort has been dedicated to enhancing these activities either by combining different EOs or adding some active compounds [34,35,36,37]. In the present study, antifungal activities of EOs and HYSs obtained from oregano (*O. vulgare*), thyme (*T. vulgaris*), lemon (*Citrus limon*), and orange (*Citrus sinensis*), against *Botrytis cinerea* have been investigated assessing their effects on hyphal growth. In addition, preventive effects of EOs, HYSs, and their mixtures were also studied by evaluating in vivo the development of gray mold disease affecting tomato fruits. We have focused our attention on tomato (*Lycopersicum esculentum*) because it is one of the most important Solanaceous crops and gray mold affects leaves and fruits, and even during postharvest, *B. cinerea* induces severe fruit rot [38,39,40].

## 2. Results and Discussion

Essential oils are commonly obtained by different methods such as, hydrodistillation, solvent extraction, and supercritical fluids extraction [19]. In this study, steam distillation, which is the most widely used method due to its low cost [41], was used to obtain EOs and HYSs from *T. vulgaris*, *O. vulgare*, *C. sinensis*, and *C. limon*. The yields of EOs were in the range of 0.50–1.75% mL/in a 100 g of dry vegetal material basis.

### 2.1. In Vitro Antifungal Activity of EOs and HYSs

**Figure 1:**
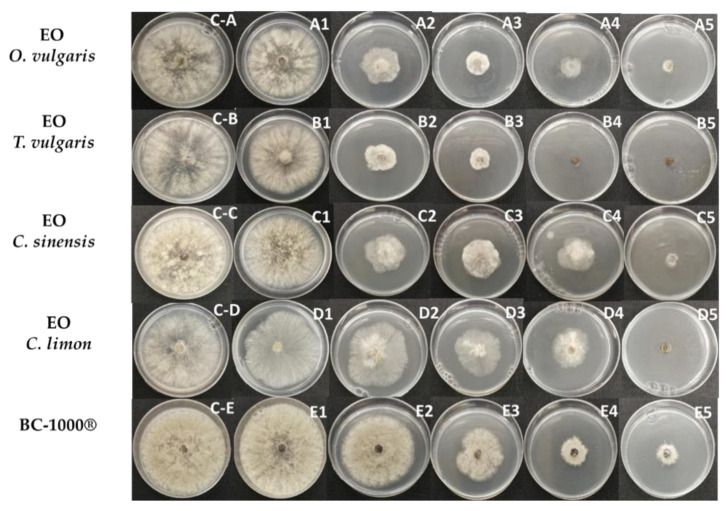
Effect of EOs, obtained from *O. vulgare*, *T. vulgaris*, *C. sinensis*, and *C. limon*, on mycelial growth of *B. cinerea* in PDA. EOs and positive control (BC-1000^®^ Dust) were applied at different concentrations (%*v*/*v*): (**C-A**–**C-E**) (negative control), 0.0; (**A1**–**E1**) 9 × 10^−4^; (**A2**–**E2**) 9 × 10^−3^; (**A3**–**E3**) 2.2 × 10^−2^; (**A4**–**E4**) 4.5 × 10^−2^; (**A5**–**E5**) 11 × 10^−2^.

**Figure 2:**
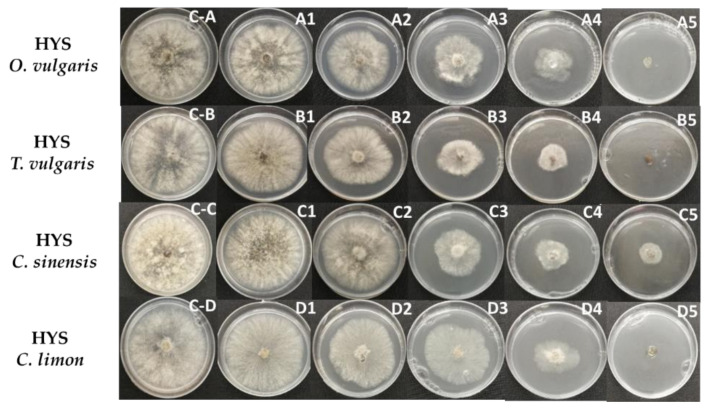
Effect of HYSs, obtained from *O. vulgare*, *T. vulgaris*, *C. sinensis*, and *C. limon*, on mycelial growth of *B. cinerea* in PDA. HYSs were added in aqueous solution at different concentrations (%*v*/*v*): (**C-A**–**C-E**) (negative control), 0.0; (**A1**–**D1**) 0.02; (**A2**–**D2**) 0.2; (**A3**–**D3**) 1.0; (**A4**–**D4**) 10; (**A5**–**D5**) 25.

The antifungal activities of EOs and HYSs were evaluated by measuring mycelial growth inhibition of *B. cinerea*. Radial growth of *B. cinerea* mycelium in potato dextrose agar (PDA) in the absence and presence of different concentrations (%*v*/*v*) of EOs and HYSs, obtained from *O. vulgare*, *T. vulgaris*, *C. sinensis*, and *C. limon*, are shown in Figure 1 and Figure 2, respectively.

The values of growth inhibition percentage induced by EOs and HYSs for all studied essential oils are given in Table 1 and Table 2, respectively.

The results obtained from the inhibition test in PDA show that the effects of both EOs and HYSs are dose dependent. In addition, the data listed in Table 1 and Table 2 indicate that EOs of *O. vulgare* and *T. vulgaris* are the most active for controlling *B. cinerea* growth. The fungus growth is completely inhibited at concentrations from 4.5 × 10^−2^ to 6.7 × 10^−2^%*v*/*v* (from 400 to 600 ppm). Similar results have been previously reported for *T. vulgaris* [42] and other species of oregano and thyme, i.e., *T. glandulosus* and *O. compactum* [43], and *T. capitatus* [44]. This inhibitory effect has been attributed to the presence of thymol and/or carvacrol [25]. These two phenolic isomer compounds are major constituents of thyme and potential application of this EO in the food industry due to its antioxidant and antimicrobial properties conferred by carvacrol and thymol have been recently reviewed [45].

To quantify the growth inhibition effect, the concentration needed to inhibit 50% of the pathogen was determined, namely the EC_50_ value. The EC_50_ values were obtained by fitting the measured percentage of inhibition to a dose-response function, and therefore they better represent the variation of inhibition with the dose of active compound. The EC_50_ values for growth inhibition of *B. cinerea* by EOs and HYSs are given in Table 3.

The data in Table 3 indicate that *T. vulgaris* and *O. vulgare* EOs are the most efficient inhibitors of mycelial growth in this study, with EC_50_ values of 15.9 and 19.8 ppm, respectively. These values are very similar to those determined for thymol and carvacrol [43]. Considering these results, and even though all studied EOs have been previously described, we determined the compositions of these two EOs (Table 4). It is worth mentioning that thymol and carvacrol were found in *T. vulgaris* EO, whereas only thymol was detected in *O. vulgare* EO. This result suggests that most of the antifungal activity observed for these EOs is due to these two isomers.

In addition, in vitro inhibition of hyphal growth of *B. cinerea* by HYSs has also been assessed. It is well known that, unlike essential oils, hydrolates are water-miscible phases in which a complex mixture of oxygenated compounds, such as alcohols, ketones, ethers, aldehydes, and esters, and very low percentages of terpenes are dissolved [46]. Therefore, HYS-induced inhibition is due to low concentrations of active compounds having enhanced diffusion in the aqueous medium. The data obtained for HYSs (Table 2 and Table 3) indicate that HYS *C. sinensis* is the most active, and its EC_50_ value is 3 to 10 times lower than that measured for the HYSs of the other EOs. This result suggests that polar compounds of *C. sinensis* are more active or are in higher concentrations than in the other studied EOs. A comparison of EO and HYS activities, measured by the EC_50_ values, show that EO activities are much higher than those observed for the respective HYSs, namely the EC_50_(HYS)/EC_50_(EO) ratio is approximately 13 for *O. vulgare* and *T. vulgaris*, 4 for *C. limon*, and 1 for *C. sinensis*. These results confirm that the main activity of *O. vulgare* and *T. vulgaris* EOs against *B. cinerea* is due mainly to nonpolar volatile compounds, whose concentrations are higher in the EO phase than in the polar HYSs. However, this difference is almost nil for *C. sinensis* suggesting that in this HYS there are polar compounds with significant activity against *B. cinerea*, and in addition, the amount of active volatile compounds in this EO is much lower than in *O. vulgare* and *T. vulgaris* EOs.

### 2.2. Chemical Composition of Essential Oils of T. vulgaris and O. vulgare

The chemical composition of EOs was determined by GC-MS (Figures S1 and S2, Supplementary Materials) and the results are in line with previous reports [42,47,48,49]. The major compounds of *T. vulgaris* EO were thymol (56.62%), *p*-cymene (17.52%), and carvacrol (5.11%), whereas *O. vulgare* EO contains mainly γ-terpinene (32.48%), α-terpineol (18.35%), terpinen-4-ol (19.01%), and thymol (5.45%). The assignments of chemical structure, presented in the chromatograms (Figures S1 and S2) and in Table 4, were obtained by comparing the mass spectra of each peak with those of the NIST11.lib database, provided by the software equipment. Additionally, the values obtained for the detected ions (*m*/*z*) were compared with reference data (Table S1) [50].

### 2.3. In Vivo Assays of Antifungal Activities of EO/HYS Mixtures and HYSs

**Figure 3:**
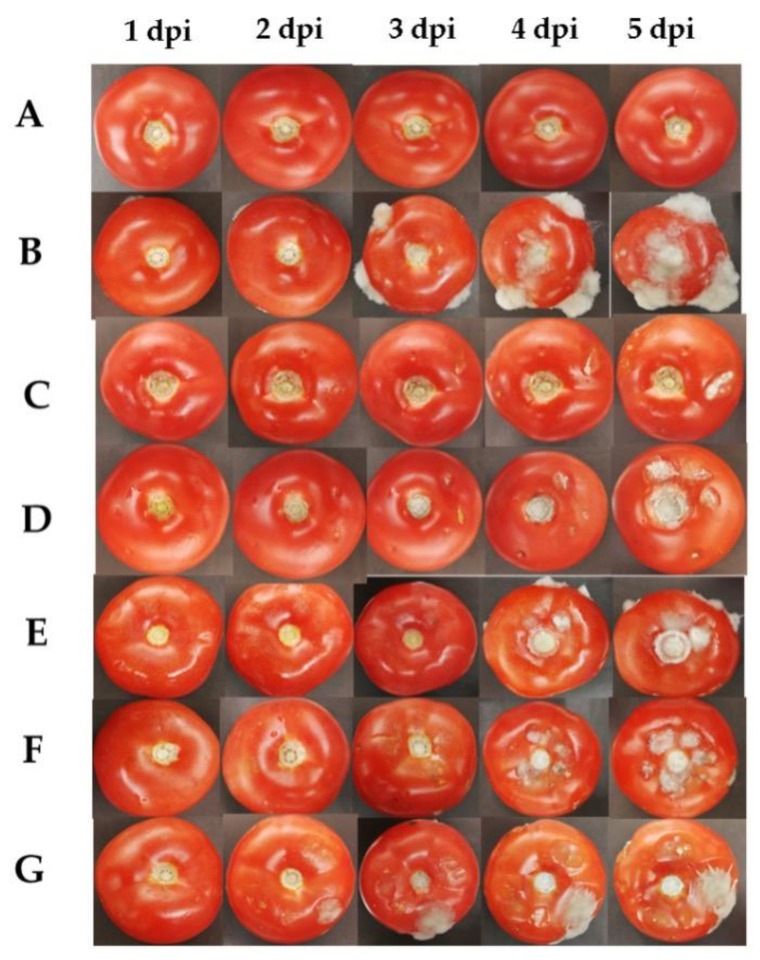
Effects of mixtures of (EO/HYS) and hydrolates (HYSs) of *T. vulgaris* and *O. vulgare* at different concentrations for controlling gray mold caused by *B. cinerea* on tomato fruits for five days post inoculation (dpi): (**A**) Control (-); (**B**) inoculum Control; (**C**) EO/HYS 0.135/30 (%*v*/*v*), thyme; (**D**) HYS 30%*v*/*v*, thyme; (**E**) EO/HYS 0.135/30 (%*v*/*v*), oregano; (**F**) HYS 30%*v*/*v*, oregano; (**G**) BC-1000^®^ 0.135%*v*/*v*.

In vivo activities of the most active EO and HYS compounds were assessed using ripe and healthy tomato fruits, which were previously sterilized and wounded. EO/HYS mixtures and HYSs of *T. vulgaris* and *O. vulgare* were sprayed on the wounds, and then a conidial suspension of *B. cinerea* was applied to inoculate the wounded fruits. Daily photographs of fruits submitted to different treatments are shown in Figure 3.

A visual inspection of results shown in Figure 3 indicate that, in the negative control, the first symptoms of gray mold disease appear two days after inoculation, whereas with EO/HYS mixtures and HYSs from thyme and oregano, the fungal infection is noticed 4 and 5 days after infection, respectively. A comparison of treatments with EO/HYS mixtures (rows C and E) and HYSs (rows D and F) indicates that thyme (rows C and D) is slightly more active than oregano (rows E and F), and definitely more effective than BC-1000^®^, the organic fungicide control.

To quantify these effects, the disease incidence percentages (DIP) were calculated [51], and the results obtained over five days after inoculation are given in Table 5.

The results obtained for the inoculum control show that *B. cinerea* infects tomato wounds very quickly, i.e., 10% at Day 1, 50% at Day 2, and 100% at Day 3 after inoculation. Treatment with the HYS/EO thyme mixture reduces these numbers to 0%, 10%, 30%, and 60% from 1 to 4 dpi, respectively. On Day 5 after inoculation, all tomato fruits become infected. Interestingly, applications of HYSs and the EO/HYS mixtures produce similar protecting effects, even though the in vitro EO growth inhibition effect concentration of the most active compounds is lowest in HYSs. It is possible that the application method determines the efficiency of adsorption of volatile compounds on the fruit surface, and consequently their antifungal activity. This result suggests that the germination process of spores inoculated on tomato surface is reduced, but hyphal growth is not affected by the presence of active compounds from EOs and/or HYSs.

**Figure 4:**
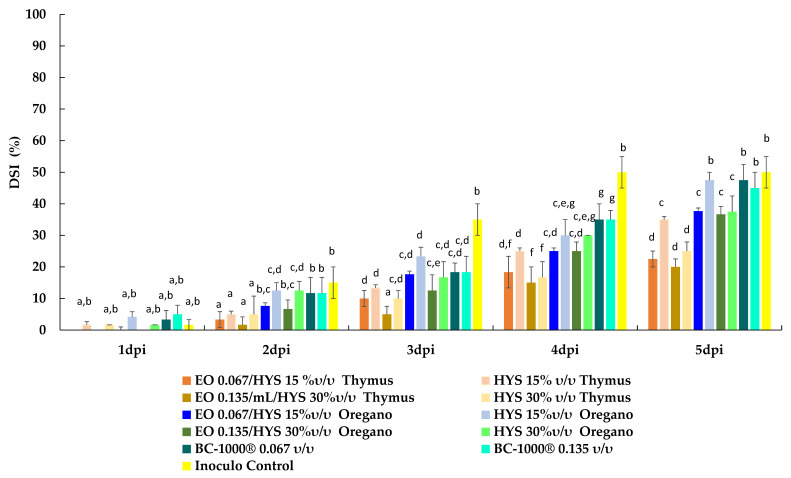
Disease severity index (%DSI) on tomato fruits measured for five days post inoculation (dpi). Values are expressed as mean ± SD. Means with the same letter are not significantly different at *p* ≤ 0.05 using the LSD test.

The disease severity index (DSI) was evaluated using a visual scale and calculated with the Townsend–Heuberger equation [52]. The results are presented in Figure 4 as the percentage of damaged fruits for each dpi. Interestingly, the highest value of DSI (50%) was reached four days after inoculation by the inoculum control, and all assayed treatments show a reduction on this index. The highest decrease in DSI is obtained for fruit treated with the EO/HYS mixture obtained from thyme.

In summary, thyme EOs and HYSs exhibit the highest antifungal activities against *B. cinerea* inoculated on tomato fruit. The lowest values of both DIP and DSI were obtained for the EO/HYS thyme mixture at concentrations 13.5 × 10^−2^/30 (%*v*/*v*). It is worthwhile to emphasize that HYSs account for a significant part of these effects. For example, this EO/HYS mixture induces a reduction of 70% and 76% in DPI and DSI, measured four days after inoculation, whereas HYSs alone reduce these indexes by 25% and 30%, respectively, as compared with the inoculum control. Similar results are found for HYSs and the EO/HYS mixture from oregano, even though the magnitude of the index reduction is lower than that determined for thyme.

## 3. Materials and Methods

### 3.1. Plant Material

*Origanum vulgare* and *Thymus vulgaris* used in this study are herbaceous plants belonging to the *Lamiaceae* family. Oregano plants were collected at the flowering state, in 2019, from Olmué, Valparaíso, Chile. Thyme plants were collected at the vegetative state, in March 2019, from El Manzano, San José de Maipo, XIII region, Chile. The foliage materials of both plants were dried at room temperature (23 °C) before the extraction process. The fruits of *Citrus limon* and *Citrus sinensis* of the Rutaceae family were collected, in September 2019, from plants cultivated at Olmué, Valparaíso, Chile.

### 3.2. Extraction of Essential Oils (EOs) and Hydrolates (HYSs)

All essential oils were obtained through the steam distillation method. Dry leaves of *T. vulgaris*, *C. limon*, and *C. sinensis* (400 g) were crushed and placed in a 2 L distillation flask designed by LABSUN. The plant material was covered with 1.3 L of distilled water, and the distillation process was maintained for 2 h. Finally, the essential oils were separated from the hydrolates by means of a burette. In the case of *O. vulgare*, the leaves (450 g) were placed in the slit of the distillation tower and submitted to stream distillation for 3 h. The essential oils and hydrolates were separated through a burette. Both essential oils and hydrolates were stored separately in amber bottles at 4 °C.

### 3.3. Fungal Material

The *B. cinerea* isolate was obtained from infested orchard vine grapes, and grown on dextrose potato agar (PDA) for 1 week at 23 °C. The pure culture was stored in the pathogen collection of the Biological Testing Laboratory of the Chemistry Department of Universidad Técnica Federico Santa María. Conidial suspension of 1 × 10^6^ conidia/mL was obtained by scraping a 10-day culture in sterile 0.05% Tween 20 aqueous solution using a hematocytometer.

### 3.4. Preparation of Essential Oil (EO) and Hydrolate (HYS) Solutions

Essential oil (200 µg) was mixed with water and Tween 20, to obtain the EO stock solution of 0.45%*v*/*v* (4000 ppm), whereas hydrolates were mixed with water and Tween 20, to obtain the HYS stock solution of 30%*v*/*v*.

The EO/HYS mixtures were prepared by mixing essential oil (200 µg) with HYS stock solution to obtain EO/HYS 0.45/30%*v*/*v*.

### 3.5. In Vitro Mycelial Growth Inhibition of Essential Oils and Hydrosols

#### Agar Dilution Method

Antifungal activity was determined using the agar dilution method, according to the method previously described [15]. The solutions were used, as previously described, to obtain different concentrations of EOs (0.009 × 10^−2^–11 × 10^−2^%*v*/*v*) and HYSs (0.02–25%*v*/*v*). The same concentrations of the positive control BC-1000^®^ Dust, CHEMIE (commercial organic fungicide), were used. The tests were carried out in 50 mm diameter petri dishes adding PDA mixed with EO, HYS, and BC-1000^®^ Dust at the concentrations and volumes indicated. The fungus was seeded by placing a 4 mm diameter inoculum disk in the center of each petri dish and allowed to incubate for 3 days at 23 °C. A negative control was performed only with the PDA culture medium. Three replicates were made for each treatment. After 3 days, the growth halo of the pathogen was measured to calculate the percentage of mycelial inhibition (%I) as compared with the control. Additionally, the effective concentration that inhibited the growth of the mycelium by 50% (EC_50_) was obtained by adjusting %I and the concentration to a dose-response equation. The fit analysis was performed using OriginPro 8.0 software (Origin Lab Corporation, Northampton, MA, USA).

### 3.6. In Vivo Antifungal Activity of HYSs and Mixtures of EO/HYS In Vivo against Gray Mold on Tomato Fruits

**Figure 5:**
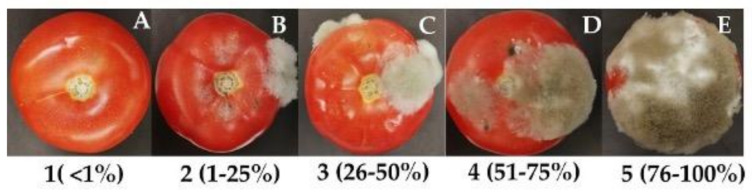
Visual scale (1–5) of symptoms caused by *B. cinerea* to quantify the incidence and severity of the disease on tomatoes: (**A**) 1 = There are no visible symptoms (not infected); (**B**) 2 = 1–25% of the inoculated area is covered with a slight necrotic mycelium (mild Infection); (**C**) 3 = 26–50% of the inoculated area is covered with necrotic mycelia (moderate Infection); (**D**) 4 = 51–75% of sample are necrotic with appeared spore masses (strong Infection); (**E**) 5 = 76–100% of the necrotic tissue appears soft and decays with a fungal mass (very Severe Infection).

Tomato fruits were obtained from a commercial market, washed with plenty of running water and superficially sterilized with a 15% sodium hypochlorite solution. Then, they were rinsed with distilled water and dried with sterile absorbent paper towel. After drying, they were placed in sterile plastic boxes, and four wounds per tomato were made using a sterile syringe. HYSs (15%*v*/*v* and 30%*v*/*v*) and EO/HYS mixtures (0.067/15%*v*/*v* and 0.135/30%*v*/*v*) were applied by spraying and, immediately thereafter, tomatoes were inoculated by spraying 1 mL of spore suspension (1 × 10^6^ conidia/mL) on the wounds of each fruit. Each experiment consisted of three replicates and each replica had four tomatoes. Organic fungicide BC1000^®^ Dust was used as a positive control at the same EO concentrations. Two additional sets of fruit were prepared, in one of them only water was added, and in the other, inoculum was added. All fruits were stored at 25 °C and 95% relative humidity for 5 days. At the end of storage, the disease incidence percentage (DIP) was calculated as DIP = (number of infected lesions)/(number of assessed lesions) × 100 [51]. The disease severity index (DSI) was calculated through the Townsend-Heuberger equation [52,53]. This equation uses a visual scale of symptoms: (1), no visible symptoms (not infected); (2), 1–25% of the inoculated area is covered with a slight necrotic mycelia (mild infection); (3), 26–50% of the inoculated area is covered with necrotic mycelia (moderate infection); (4), 51–75% of sample are necrotic with appeared spore masses (strong infection); (5), 76–100% of the necrotic tissue appears soft and decays with a fungal mass (very severe infection) (Figure 5).

A nonparametric analysis of the DIS data, obtained during the five days of post-inoculation treatment, was performed with the Matlab program 2020a, (Mathworks Inc., Natick, MA, USA) and using the multi-comparison function with a least significance difference test (LSD) with a *p* ≤ 0.05 of significance.

The confirmation of infection by *B. cinerea* was carried out by isolating the pathogen from tomato lesions and the morphological characteristics of the colony and conidia were identified under the Leica EZ4HD Stereo Microscope with camera software and DM500 microscope (Leica Microsystems, Wetzlar, Germany).

### 3.7. Chromatographic Analyses of O. vulgare and T. vulgaris

The chromatographic analyses of essential oils obtained from of *O. vulgare* and *T. vulgaris* were carried out in a GCMS-QP2010 (SHIMADZU Gas Chromatograph-Mass Spectrometer, Japan), equipped with a capillary column Rtx-5MS (30 m, 0.25 mm internal diameter, 0.25 µm film thickness) and a 5971 A mass spectrometer detector. Helium was used as carrier gas, at a flow rate of 1.61 mL/min. The column temperature was initially set to 60 °C for 5 min, and gradually increased to 160 °C (5 °C/min), and finally increased to 280 °C (15 °C/min). The injector and detector temperatures were set at 250 and 280 °C, respectively. For detection by GC-MS, an electron ionization system with an ionization energy of 70 eV was used. EOs were diluted with dichloromethane (1:1000 *v*/*v*), and 1.0 µL of diluted samples were automatically injected into no division mode. The results were obtained by comparing the mass spectra of each peak with the NIST11.lib database provided by the instrument’s software. Identification was given to those peaks exceeding the 93–95% coincidence. Additionally, calibration curves of standard compounds (Thymol) were performed and used for quantification.

## 4. Conclusions

Formulations based on EOs and HYSs exhibit antifungal activity against *B. cinerea*, both in vitro and in vivo. In all the experiments, the assayed treatments were more effective than BC-1000^®^ Dust, which is a nontoxic fungicide based on grapefruit (*Citrus* × *paridisi*) seed and pulp extracts. The antimicrobial activity is due to one or more compounds present in the EOs and HYSs. Thus, in vitro inhibition of EOs is attributed mainly to the activity of volatile compounds, whereas the HYS activity is due to polar compounds that are not present in the respective EOs. The latter are especially important in *C. sinensis*, where the lowest EC_50_ value was obtained for HYSs, several times lower than that measured for *C. limon*.

Assessments of the in vivo preventive effects against *B. cinerea* were carried out using tomato fruit and EO/HYS formulations. DIP and DIS were determined five days after inoculation and the results indicate that preventive effects depend on relative concentration and nature of both EOs and HYSs. Although most tomatoes were infested at 5 dpi, the damage was considerably reduced by application of the EO/HYS mixtures. These results suggest that a combination of HYSs and EOs enhances antifungal activity, and that by varying concentrations, volumes, and the nature of the compounds it would be possible to reach a lower level of incidence and damage and could be possible to achieve complete control of *B. cinerea* inoculum on tomato fruits.

Thus, mixtures of EOs/HYSs could be developed as a potential formulation for in vivo application in agricultural disease control. It is important to emphasize that the final formulation would be completely natural, minimizing the negative environmental impact caused by synthetic fungicides, and at the same time, would not represent a risk to human health. In addition, being a product that uses HYSs as an active compound, it contributes to the development of the circular economy, giving added value to a by-product that is generally discarded.

Antifungal activities of mixtures of EOs and HYSs from different sources are currently being evaluated.

## Figures and Tables

**Table 1:** Percentage of inhibition of *B. cinerea* mycelial growth induced by different concentrations (%*v*/*v*) of *T. vulgaris*, *O. vulgare*, *C. sinensis*, and *C. limon* EOs, three days after incubation.

Concentration	* Percentage of Inhibition of Mycelial Growth of *B. cinerea* (%) ± SD				
%*v*/*v* × 10^−2^	*O. vulgare*	*T. vulgaris*	*C. sinensis*	*C. limon*	BC-1000^®^
0.009	9 ± 5.0 ^a,e^	17 ± 4.1 ^c^	2 ± 2.0 ^b,d,e^	12 ± 6.0 ^a,c^	0 ± 0.0 ^d^
0.045	30 ± 4.3 ^b,d^	24 ± 13.4 ^c,d^	14 ± 10.2 ^a,b,c^	12 ± 3.0 ^a,b,c^	0 ± 0.0 ^a^
0.09	38 ± 10.3 ^e^	36 ± 9.4 ^e^	19 ± 2.3 ^b,c^	14 ± 4.5 ^a,b^	0 ± 0.0 ^d^
0.90	59 ± 2.5 ^b,c,d^	75 ± 7.8 ^e^	56 ± 12.8 ^b,c^	25 ± 5.9 ^a^	15 ± 10.8 ^a^
2.26	80 ± 3.3 ^c,d^	83 ± 2.7 ^d^	56 ± 4.9 ^b^	52 ± 7.4 ^a,b^	39 ± 11.8 ^a^
4.52	83 ± 2.2 ^c^	96 ± 7,2 ^e^	79 ± 7.9 ^a,b^	59 ± 9.8 ^a^	77 ± 1.0 ^b,c^
6.78	100 ± 0.0 ^d^	100 ± 0.0 ^d^	73 ± 12.8 ^b,c^	66 ± 4.9 ^a,b^	71 ± 3.9 ^a,b,c^
9.0	100 ± 0.0 ^d^	100 ± 0.0 ^d^	85 ± 7.9 ^a,c^	73 ± 3.9 ^b^	78 ± 0.0 ^b,c^
11.3	100 ± 0.0 ^a^	100 ± 0.0 ^a^	88 ± 2.0 ^c^	99 ± 0.9 ^a^	83 ± 1.0 ^b^

* Values are the average of three replicates per treatment ± standard deviation. Mean values with the same superscript letter do not differ between them according to LSD test (*p* ≤ 0.05).

**Table 2:** Percentage of inhibition of *B. cinerea* mycelial growth induced by HYSs of *T. vulgaris*, *O. vulgare*, *C. sinensis*, and *C. limon* at different concentrations (%*v*/*v*) three days after incubation.

Concentration	* Percentage of Inhibition of Mycelial Growth of *B. cinerea* (%) ± SD				
%*v*/*v*	*O. vulgare*	*T. vulgaris*	*C. sinensis*	*C. limon*	BC-1000^®^
0.02	12 ± 1.3 ^a.c^	15 ± 2.1 ^a,c^	0 ± 0.0 ^d^	9 ± 9.9 ^a,b^	0 ± 0.0 ^d^
0.1	25 ± 4.5 ^b,c,d^	26 ± 14.5 ^c,d^	11 ± 7.9 ^a,b^	14 ± 2.0 ^a,b,c^	0 ± 0.0 ^a^
0.2	25 ± 2.5 ^c^	26 ± 3.7 ^c^	8 ± 6.0 ^a,d^	18 ± 3.4 ^a,b,c^	0 ± 0.0 ^d^
1	47 ± 1.3 ^b,c^	67 ± 12.2 ^c,d,e^	50 ± 7.9 ^b^	22 ± 3.0 ^a^	15 ± 10.8 ^a^
5	62 ± 16.3 ^b^	64 ± 16.6 ^b,c^	56 ± 9.3 ^a,b^	39 ± 5.9 ^a^	39 ± 11.8 ^a^
10	64 ± 10.3 ^a,d^	75 ± 0.0 ^b,c,d^	67 ± 9.7 ^a,b^	59 ± 1,1 ^a^	77 ± 1.0 ^b,c^
15	72 ± 3.3 ^b,c^	79 ± 15.3 ^c^	80 ± 1.1 ^c^	60 ± 3.0 ^a^	71 ± 3.9 ^a,b,c^
20	83 ± 8.2 ^a,c^	82 ± 0.0 ^a,c^	88 ± 5.7 ^a^	86 ± 0.0 ^a^	78 ± 0.0 ^b,c^
25	100 ± 0.0 ^a^	92 ± 5.5 ^c^	84 ± 3.9 ^b^	100 ± 0.0 ^a^	83 ± 1.0 ^b^

* Values are the average of three replicates per treatment ± standard deviation. Mean values with the same superscript letter do not differ between them according to LSD test (*p* ≤ 0.05).

**Table 3:** EC_50_ values calculated for inhibition of mycelial growth of *B. cinerea* in vitro. Measurements were performed three days after incubation.

Antifungal Agent	EO, %*v*/*v* × 10^−2^ (ppm)	HYS, %*v*/*v*
*O. vulgare*	2.2 ± 0.07 ^f^ (19.8)	2.5 ± 0.8 ^b,c,d^
*T. vulgaris*	1.8 ± 0.06 ^f^ (15.9)	2.1 ± 0.7 ^a,b,c,d^
*C. sinensis*	9.4 ± 0.06 ^d^ (83.2)	0.7 ± 0.9 ^a,b^
*C. limon*	22.2 ± 0.06 ^b^ (196.2)	7.6 ± 0.9 ^e^
BC-1000^®^ (C+)	270.8 ± 0.02 ^a^	
Carvacrol	(18.6) *	
Thymol	(18.9) *	
*O. compactum*	(35.1) *	
*T. glandulosus*	(79.1) *	

Same letters in superscript do not present significant differences (*p* ≤ 0.05). EC_50_ values for EOs given in parenthesis are in ppm. * Data from [43].

**Table 4:** Chemical composition of essential oils extracted from leaves of *T. vulgaris* (thyme) and *O. vulgare* (oregano) determined by gas chromatography coupled to mass detection (GC-MS).

Compounds	^a^ Percentage *T. vulgaris*	^b^ RT (min.) *T. vulgaris*	^a^ Percentage *O. vulgare*	^b^ RT (min.) *O. vulgare*
α-Terpinenol	-	-	18.35	8.859
β-Cymene	17.52	9.144	3.11	9.142
γ-Terpinene	3.30	10.331	32.48	10.334
Terpinolene	-	-	7.47	11.348
β-Linalool	3.42	11.741	5.11	11.742
Borneol	2.81	13.937	-	-
Terpinen-4-ol	-	-	19.01	14.315
α-Terpineol	-	-	2.89	14.744
Isothymol methyl ether	1.90	16.416	2.70	16.418
p-Thymol	56.62	17.919	5.45	17.879
β-Caryophyllene	-	-	3.43	21.527
Carvacrol	5.11	18.161	-	-

^a^ Percentage, determined as the ratio, i.e., peak area/area total, for each peak in the chromatogram. ^b^ RT, retention time.

**Table 5:** Effect of different treatments applied in vivo on tomato fruits on the incidence of *Botrytis cinerea* disease over five days post-inoculation (dpi).

Disease Incidence Percentage of Gray Mold Disease (%) ± SD							
Plants	Treatments	Concentration (%*v*/*v*)	1 dpi	2 dpi	3 dpi	4 dpi	5 dpi
Thyme	EO/HYS	6.7 × 10^−2^/15	0 ± 0.0 ^a^	16.7 ± 14.3 ^a,c,d^	50.0 ± 25.0 ^c^	83.3 ± 14.3 ^b,c^	91.7 ± 14.3 ^b^
	HYS	15	8.3 ± 14.3 ^a,b^	25 ± 25.0 ^a,c,d^	58.3 ± 28.6 ^b,c^	75 ± 25.0 ^c,d^	100 ± 0.0 ^b^
	EO/HYS	13.5 × 10^−2^/30	0 ± 0 ^a^	8.3 ±14.3 ^a,c^	33.3 ± 28.6 ^a^	58.3 ± 14.3 ^d^	91.7 ± 14.3 ^b^
	HYS	30	8.3 ± 14.3 ^a,b^	25 ± 25.0 ^a,c,d^	33.3 ± 14.3 ^a^	75 ± 14.3 ^c,d^	91.7 ± 14.3 ^b^
Oregano	EO/HYS	6.7 × 10^−2^/15	0 ± 0 ^a^	33.3 ± 12.5 ^a,b^	87.8 ± 12.5 ^b^	100.0 ± 0.0 ^b^	100.0 ± 0.0 ^b^
	HYS	15	16.7 ± 28.8 ^a,b^	66.7 ± 14.3 ^b^	83.3 ±14.3 ^b,c^	91.7 ± 14.3 ^b,c^	100.0 ± 0.0 ^b^
	EO/HYS	13.5 × 10^−2^/30	0 ± 0 ^a^	33.3 ± 28.8 ^a,b^	75.0 ± 25.0 ^b,c^	91.7 ± 14.3 ^b,c^	100.0 ± 0.0 ^b^
	HYS	30	8.3 ± 14.3 ^a,b^	33.3 ± 1.3 ^a,b^	66.7 ± 25.0 ^b,c^	91.7 ± 14.3 ^b,c^	100.0 ± 0.0 ^b^
C (+)	BC-1000^®^	6.7 × 10^−2^	16.7 ± 14.3 ^b^	58.3 ± 14.3 ^b,d^	83.3 ± 14.3 ^b,c^	100.0 ± 0.0 ^b^	100.0 ± 0.0 ^b^
		13.5 × 10^−2^	25.0 ± 0.0 ^a,b^	50.0 ± 0.0 ^b,d^	75.0 ± 25.0 ^b,c^	100.0 ± 0.0 ^b^	100.0 ± 0.0 ^b^
Control	Inoculum	-	8.3 ± 14.4 ^a,b^	41.7 ± 52.0 ^b^	83.3 ± 28.8 ^b,c^	100 ± 0.0 ^b^	100 ± 0.0 ^b^
	Water	-	0 ± 0.0 ^a^	0 ± 0.0 ^a^	0 ± 0.0 ^a^	0 ± 0.0 ^a^	0 ± 0.0 ^a^

C (+), positive control; Same letters in superscript do not present significant differences (*p* ≤ 0.05).

## Data Availability

Not applicable.

## Supplementary Materials

The following are available online at https://www.mdpi.com/article/10.3390/plants10081719/s1, Figure S1: Chromatogram GC-MS of essential oil obtained from *Thymus vulgaris*, and chemical structures of identified compounds, Figure S2: Chromatogram GC-MS of essential oil obtained from *Oreganum vulgare* (oregano), and chemical structures of identified compounds, Table S1: Retention times and quantification ions of compounds present in essential oils extracted from leaves of *T. vulgaris* (thyme) and *O. vulgare* (oregano) using gas chromatography coupled to mass detection (GC-MS).
