# Supplementary material for: Assessing the Control of Postharvest Gray Mold Disease on Tomato Fruit Using Mixtures of Essential Oils and Their Respective Hydrolates
Source: Plants (Basel). 2021 Aug 20;10(8):1719. doi: 10.3390/plants10081719 (PMC8401415; doi:10.3390/plants10081719)
Supplement: Supplementary file 1 [file plants-10-01719-s001.zip › plants-1337191-supplementary.pdf]

plants

Article

Assessing the Control of Postharvest Gray Mold Disease on Tomato Fruit Using Mixtures of Essential Oils and Their Respective Hydrolates

Conny Brito [1], Henrik Hansen [1], Luis Espinoza [2], Martín Faúndez [2], Andrés F. Olea [3,*], Sebastián Pino [4] and Katy Díaz [2,*]

[1] Departamento de Ingeniería Química y Ambiental, Universidad Técnica Federico Santa María, Avenida España 1680, Valparaíso 2340000, Chile; connybritoescudero@gmail.com (C.B.); Henrik.hansen@usm.cl (H.H.)
[2] Departamento de Química, Universidad Técnica Federico Santa María, Avenida España 1680, Valparaíso 2340000, Chile; luis.espinozac@usm.cl (L.E.); martin.faundez.12@usm.cl (M.F.)
[3] Instituto de Ciencias Químicas Aplicadas, Facultad de Ingeniería, Universidad Autónoma de Chile, El Llano Subercaseaux 2801, Santiago 8900000, Chile
[4] LABSUN (Laboratorio Sustentable Natural), Valparaíso 2340000, Chile; sebastian.pino@alumnos.usm.cl
* Correspondence: andres.olea@uautonoma.cl (A.F.O.); katy.diaz@usm.cl (K.D.); Tel.: +56-32-2652843 (K.D.)

Supplementary Materials: The following are available online at https://www.mdpi.com/article/10.3390/plants10080000/s1, Figure S1: Chromatogram GC-MS of essential oil obtained from Thymus vulgare, and chemical structures of identified compounds; Figure S2: Chromatogram GC-MS of essential oil obtained from Oreganus vulgare (Oregano), and chemical structures of identified compounds; Table S1: Retention times and quantification ions of compounds present in essential oils extracted from leaves of T. vulgaris (Thymus) and O. vulgare (Oregano) using Gas Chromatography coupled to Mass Detection (GC-MS).

Citation: Brito, C.; Hansen, H.; Espinoza, L.; Faúndez, M.; Olea, A.F.; Pino, S. Assessing the Control of Postharvest Gray Mold Disease on Tomato Fruit Using Mixtures of Essential Oils and Their Respective Hydrolates. *Plants* **2021**, *10*, 1719. https://doi.org/10.3390/plants10081719

Academic Editor: Stefania Garzoli

Received: 27 July 2021
Accepted: 14 August 2021
Published: 20 August 2021

Publisher's Note: MDPI stays neutral with regard to jurisdictional claims in published maps and institutional affiliations.

Copyright: © 2021 by the authors. Submitted for possible open access publication under the terms and conditions of the Creative Commons Attribution (CC BY) license (http://creativecommons.org/licenses/by/4.0/).

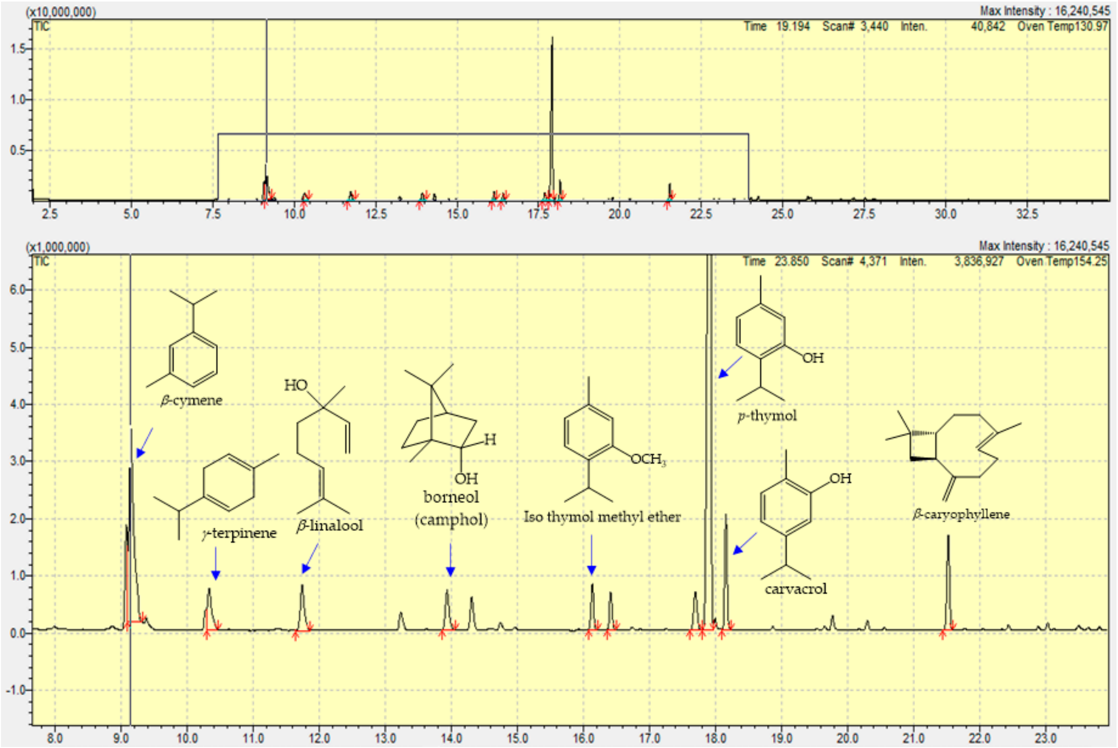

Figure S1. Chromatogram GC-MS of essential oil obtained from Thymus vulgare (Thymus), and chemical structures of identified compounds.

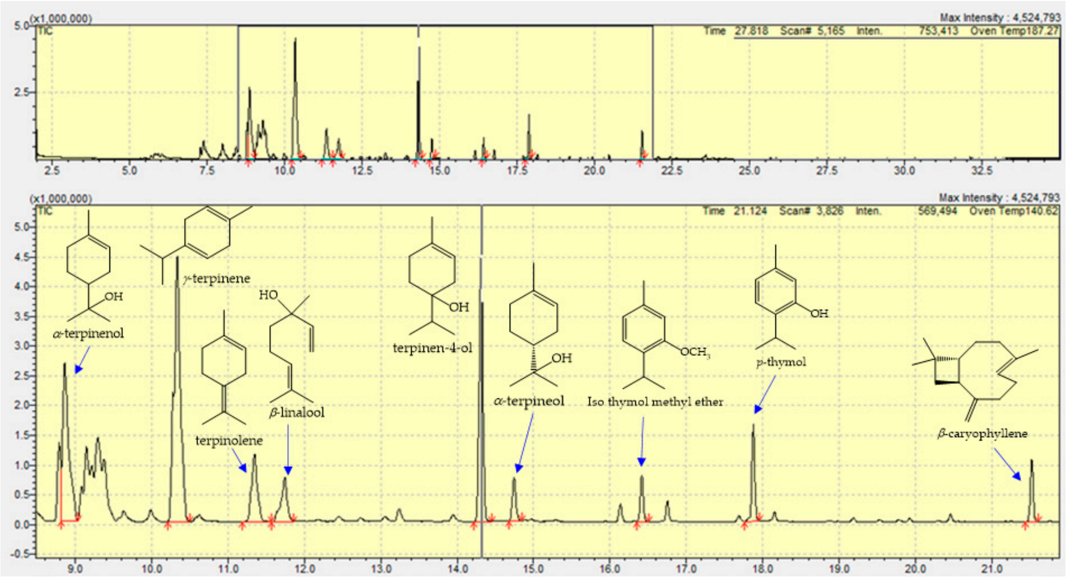

Figure S2. Chromatogram GC-MS of essential oil obtained from Oreganus vulgare (Oregano), and chemical structures of identified compounds.

Table S1. Retention times and quantification ions of compounds present in essential oils, extracted from leaves of T. vulgaris (Thymus) and O. vulgare (Oregano), using Gas Chromatography coupled to Mass Detection (GC-MS).

Compounds	[a]RT (min.) T. vulgaris	RT (min.) O. vulgare	[b]Area (%) T. vulgaris	Area (%) O. vulgare	peak Identification (m/z)
α-Terpinenol	-	8.859	-	18.35	MS (154)
β-Cymene	9.144	9.142	17.52	3.11	MS (134)
γ-Terpinene	10.331	10.334	3.30	32.48	MS (136)
Terpinoleno	-	11.348	-	7.47	MS (136)
β-Linalool	11.741	11.742	3.42	5.11	MS (154)
Borneol	13.937		2.81		MS (154)
Terpinen-4-ol	-	14.315	-	19.01	MS (154)
α-Terpineol	-	14.744	-	2.89	MS (154)
Isothymol methyl ether	16.416	16.418	1.90	2.70	MS (164)
p-Thymol	17.919	17.879	56.62	5.45	MS (150)
β-Caryophylle	-	21.527	-	3.43	MS (204)
Carvacrol	18.161	-	5.11	-	MS (150)
Caryophyllene	21.527	4.53	-	-	MS (204)

[a]RT, retention time; [b]Area, relative amount of the identified compounds based on the area of each peak in the total area of the chromatogram.
